# Editorial on the Special Issue “Heme Metabolism and Porphyria”

**DOI:** 10.3390/life14050581

**Published:** 2024-04-30

**Authors:** Elena Di Pierro, Jasmin Barman-Aksözen, Emmanuel Richard

**Affiliations:** 1 Fondazione IRCCS Ca’ Granda Ospedale Maggiore Policlinico, 20122 Milan, Italy; 2 Institute of Laboratory Medicine, Municipal Hospital, 8063 Zurich, Switzerland; jasmin.barman@triemli.zuerich.ch; 3 Medical Science Faculty, University of Bordeaux, 33076 Bordeaux, France; emmanuel.richard@u-bordeaux.fr; 4 Service de Biochimie, Laboratoire de Biologie Médicale de Référence (LBMR), Anomalies du Métabolisme de L’hème, Groupe Hospitalier Pellegrin, 33000 Bordeaux, France; 5 Centre de Compétence Maladies Rares Porphyries et Anémies Rares du Métabolisme du fer, 33000 Bordeaux, France

Porphyria denotes a heterogeneous group of metabolic disorders caused by anomalies in the biosynthesis of heme, a crucial component of hemoglobin and other vital hemoproteins. Porphyria is classified into acute and cutaneous types; acute forms are distinguished by severe acute neurovisceral attacks, whereas the cutaneous forms are characterized by skin blistering and sensitivity to light [1,2,3].

In recent years, significant efforts have been devoted to enhancing our understanding of these rare disorders. Despite these advancements, due to their complexity and heterogeneous clinical presentations, porphyrias continue to pose diagnostic challenges for many healthcare professionals. Furthermore, certain aspects of their pathogenesis have yet to be fully elucidated, and effective treatment remains a significant unmet need. This is because existing medications do not completely correct the heme biosynthesis pathway and are not available for all forms of the disease [4,5,6].

This Special Issue, titled “Heme Metabolism and Porphyria”, aims to bring to light recent developments in both basic and translational research on acute and cutaneous porphyrias. It includes ten contributions, with nine research articles and one review.

Among these, an original study offers insights into the differences between the mitochondrial heme metabolon in erythroid and non-erythroid cells, suggesting new avenues for understanding the differential regulation of heme synthesis and homeostasis in these cells [7]. Moreover, the interaction of human frataxin and ferrochelatase (FECH) with protoporphyrin IX (PPIX), which facilitates heme biosynthesis, is explored as a potential post-translational regulatory mechanism [8].

New perspectives on the pathogenesis of Acute Intermittent Porphyria (AIP) have also emerged. The discovery of a first de novo mosaic mutation causing acute porphyria symptoms highlights the utility of third-generation long-read single-molecule sequencing as a diagnostic tool in cases where pathogenetic variants cannot be identified using standard molecular methods [9]. Additionally, the finding of a reduced number of mitochondria in AIP patients, alongside a significant correlation between disease penetrance and reductions in mitochondrial DNA (mtDNA) copy number and markers of mitochondrial biogenesis, offers a novel explanation for the clinical variability observed in acute porphyria [10].

The investigations included in this Special Issue also extend to the evaluation of modern serum and urine biomarkers for monitoring renal and hepatic damage and to the early detection of kidney and liver injury in AIP [11]. Moreover, the association between organ complications, chronic low-grade inflammation, insulin resistance, and poorer dental health in AIP patients is described for the first time [12].

Regarding cutaneous porphyria, a report on a French cohort of perinatal cases of Congenital Erythropoietic Porphyria (CEP) emphasizes the clinical heterogeneity and progression of the disease, in addition to the increasing number of cases encountered worldwide. CEP, which is among the rarest and most severe porphyrias, underscores the critical importance of early diagnosis and timely therapeutic intervention as well as the need for novel treatment approaches [13].

This Special Issue also presents findings that suggest how treatment strategies developed over the past four decades, alongside increased disease awareness and the prevention of new attacks through family counselling, have reduced mortality rates in AIP pedigrees, leading to life expectancies comparable to those in the general population and improved prognoses thanks to lifestyle modifications [14]. Additionally, a study on a larger cohort of Erythropoietic Protoporphyria (EPP) patients reveals a specific protective effect of afamelanotide against EPP-related liver dysfunction and PPIX accumulation, indicating a dose-dependent action of Scenesse^®^ in the pathophysiological process of EPP besides its known role in skin photoprotection, thus providing new insights for medical decisions regarding drug dosage [15].

Identifying curative treatments for both hepatic and cutaneous porphyria, however, remains a challenge. An update on novel and established treatments for AIP is provided, highlighting the potential of emerging mRNA transfer technology. Its efficacy in enhancing PBGD activity in the liver and in restoring the physiological regulation of the heme pathway has been confirmed in both mouse and large-animal studies [16].

In summary, this collection offers further insights into the pathophysiology, genetics, diagnosis, and treatment of porphyrias, opening new research avenues and underscoring the importance of continued research in the field of rare diseases.
